# Symbolic Signal Use in Wild Chimpanzee Gestural Communication?: A Theoretical Framework

**DOI:** 10.3389/fpsyg.2021.718414

**Published:** 2021-12-24

**Authors:** Julia Cissewski, Lydia V. Luncz

**Affiliations:** ^1^Department of Human Behavior, Ecology, and Culture, Max Planck Institute for Evolutionary Anthropology, Leipzig, Germany; ^2^Technological Primates Research Group, Max Planck Institute for Evolutionary Anthropology, Leipzig, Germany

**Keywords:** symbolic communication, great apes, chimpanzees, gestures, arbitrariness, conventionalization

## Abstract

Symbolic communication is not obvious in the natural communicative repertoires of our closest living relatives, the great apes. However, great apes do show symbolic competencies in laboratory studies. This includes the understanding and the use of human-provided abstract symbols. Given this evidence for the underlying ability, the apparent failure to make use of it in the wild is puzzling. We provide a theoretical framework for identifying basic forms of symbolic signal use in chimpanzee natural communication. In line with the laboratory findings, we concentrate on the most promising domain to investigate, namely gesture, and we provide a case study in this area. We suggest that evidence for basic symbolic signal use would consist of the presence of two key characteristics of symbolic communication, namely arbitrariness and conventionalization. Arbitrariness means that the linkage between the form of the gesture and its meaning shows no obvious logical or otherwise motivated connection. Conventionalization means that the gesture is shared at the group-level and is thus socially learned, not innate. Further, we discuss the emergence and transmission of these gestures. Demonstrating this basic form of symbolic signal use would indicate that the symbolic capacities revealed by laboratory studies also find their expression in the natural gestural communication of our closest living relatives, even if only to a limited extent. This theoretical article thus aims to contribute to our understanding of the developmental origins of great ape gestures, and hence, arguably, of human symbolic communication. It also has a very practical aim in that by providing clear criteria and by pointing out potential candidates for symbolic communication, we give fieldworkers useful prerequisites for identifying and analyzing signals which may demonstrate the use of great apes’ symbolic capacities in the wild.

## Introduction

Symbolic communication is still regarded as a capacity that separates humans from other animals (e.g., Deacon, 2012), thus making us the “symbolic species” (Deacon, 1997). And it is true that language, which is a highly complex, multi-level system of symbolic communication (Deacon, 1997; Webster, 2017), can be found only in humans.

One should not conclude from this, however, that symbolic signal use is absent in other animals’ natural communicative repertoires. In order to detect cases of symbolic signal use in other species, we propose avoiding a language-centered approach and concentrating instead on the basic characteristic of symbolic communication: the arbitrary and conventionalized linkage between the symbol’s form (e.g., sound shape) and its meaning (concept). This follows Saussure’s *arbitrariness of the sign*, that is, the distinction between *le signifiant (the signifier)* and *le signifié (the signified*; de Saussure, 1916). *Arbitrariness* is also one of Hockett’s design features of language (Hockett, 1960).

The form-meaning linkage of the symbol is “arbitrary,” because there is no logical or otherwise motivated connection between form and meaning. Thus, the word *book* (in its spoken or written form) does not resemble the object that it denotes. Importantly, this arbitrary linkage is not genetically determined, but is transmitted socially and is thus “conventionalized” among the members of a group (e.g., Chandler, 2017; Crystal, 2019), in this case a language community. Thus, to stay with the example, different languages use different words (for instance, *book*, *livre*, and *книга*) for the same object. The presence of communicative signals with these two characteristics, namely arbitrary form-meaning linkage and conventionalization, in non-human animals’ natural communication would thus provide evidence for the existence of basic symbolic signal use. In the section “Criteria for basic symbolic signal use” we describe the application of these two characteristics to chimpanzee natural gestural communication. We emphasize the very basic nature of these criteria compared to Deacon’s definition of human symbolic representation.

Intriguingly, studies in laboratory settings have revealed symbolic capacities in our closest living relatives, the great apes (e.g., Patterson, 1978; Savage-Rumbaugh et al., 1986; Greenfield and Savage-Rumbaugh, 1990; Miles, 1990), as well as in marine mammals (e.g., Schusterman and Krieger, 1984; Herman, 1987), dogs (e.g., Kaminski et al., 2004), and parrots (e.g., Pepperberg and Nakayama, 2016). For a general review, see Pepperberg (2017). The bonobos, chimpanzees, gorillas, and orangutans participating in these studies were able to acquire human-provided conventionalized arbitrary signs like lexigrams and gestures from American Sign Language (ASL). They learned both to understand them and to communicate with them. This included the combination of signs to form short utterances. Moreover, they showed cognitive abilities such as categorization (the mental grouping of objects, subjects etc. according to specific properties and for specific purposes) and decontextualization (the isolation and generalization of a mental representation from the original context). It should be said that not all specialists are convinced that the published literature demonstrates that captive apes are capable of symbolic communication; for examples of this critique and for a balanced review, see Pepperberg (2017). While in our view, the evidence from the studies of great apes in captivity points to the presence of symbolic competencies, these competencies are not obvious in the apes’ communicative repertoires in their natural environment.

Here, we focus on great apes’ symbolic capacities. Given the laboratory findings outside the vocal domain, we will concentrate on their natural gestural communication, as we search for evidence of symbolic signal use, and specifically in chimpanzees. Note that while symbolic communication may exist in the chimpanzees’ natural gestural repertoire, the number of potential candidates reported in this paper is rather small and largely confined to two contexts (playing and mating). Note further that in laboratory studies the concepts in the human-provided abstract symbols can be narrowed down considerably. This is not possible to the same extent in the concepts underlying the potentially symbolic gestures mentioned in this paper. Nevertheless, it is not obligatory for these gestures to be associated with very narrow concepts to qualify as arbitrary and conventionalized signals. The important criterion is that the users share these concepts as a result of group-specific conventionalization.^1^

We now turn to the natural gestural repertoires of the great apes and to the criteria for defining a basic form of symbolic communication.

### Great Ape Gestures

Gestures are an important element of great ape communication. They can be defined as intentional movements of body parts like hands, limbs, or the head, and body postures that are directed toward another individual, are goal-directed, motorically ineffective (toward the recipient), and receive a voluntary response (Tomasello and Call, 2007). Gestures are used by great apes in the wild (MacKinnon, 1974; Goodall, 1986; Genty et al., 2009; Graham et al., 2017) and in captive settings (e.g., Tomasello et al., 1989; Pika et al., 2003, 2005; Liebal et al., 2006). Examples of gestures in chimpanzees would be PRESENT BODY PART^2^ (visual modality), TOUCH (tactile modality), and STOMP (auditory modality).

Gestures in great apes are used flexibly and in accordance with the attentional state of the recipient (e.g., Liebal et al., 2004). That is, gestures of the visual modality are more likely to be employed when the recipient is attending to the sender, and tactile gestures when the recipient is not attending; the sender may visually check the attentional state of the recipient and exhibit response-waiting. The same gesture may be used in different contexts, and a single context may elicit several different gestures (e.g., Tomasello and Call, 2007).

Interestingly, great ape gestures may involve objects in the physical environment. An example for such object-associated gestures is the auditory gesture of KNUCKLE-KNOCKING found in chimpanzees of the North group of Taï National Park (Côte d’Ivoire), which consists of the knocking of knuckles on a hard surface, for instance on tree branches (Boesch, 1995). The auditory gesture of LEAF-CLIPPING that can be observed, for instance, in the chimpanzees of Mahale (Tanzania) consists of taking off parts of leaves with the mouth or fingers, thereby causing a distinctive sound (Nishida, 1980). Both auditory gestures are used for sexual solicitation in the respective group.

At the ontogenetic level, Tomasello and Call (2007, 2019) divide great ape gestures into *attention getters* and *intention movements*. *Attention getters* (e.g., GROUND SLAP) draw the attention of the audience to the sender without carrying information about the specific meaning. The recipient needs to infer this meaning from the behavior accompanying the *attention getter*. *Intention movements* are truncated forms of social behaviors (e.g., ARM RAISE as a ritualization of play hitting) and therefore do not exhibit a truly arbitrary form-meaning linkage. *Intention movements* are used in the context of the underlying social behavior and their meaningfulness normally is ensured from the context. Thus, neither *attention getters* nor *intention movements* as defined by Tomasello and Call qualify as learned arbitrary gestures.

### Criteria for Basic Symbolic Signal Use – and Some Potential Candidates

In our search for symbolic signal use in our closest living relatives, we propose to identify great ape gestures that fulfill the basic criteria of *arbitrariness* of form-meaning linkage and *conventionalization* among the members of a group, as outlined in the *Introduction*.

By *arbitrariness* of form-meaning linkage, we mean the absence of any logical or otherwise motivated connection between the form and the meaning of a gesture. Arbitrary linkage is thus different from iconic linkage (where the form resembles the meaning) and also from indexical linkage (where the link to the referent can be observed or inferred; this includes pointing). Note that for *arbitrariness* it is not necessary that the form of the gesture is abstract in the sense of, for example, Arabic numbers. Rather, it would be sufficient for the form not to resemble or not be otherwise connected to the meaning of the gesture. We will see later what the possibilities might be in the case of chimpanzee gestures.

Evidence for *conventionalization*,^3^ that is, the sharing of form-meaning linkages among individuals, should be sought in gestures that are learned, for instance, at the group level. These group-specific gestures, that is, gestures that are shared by some or most individuals in a group but are absent in other groups of the same species, strongly suggest social transmission (e.g., Bonnie and de Waal, 2006) rather than innateness. For a contrary view, see, for instance, Byrne et al. (2017). Group-specific gestures can be found in great apes in the wild (e.g., Whiten et al., 1999, 2001) and in captivity (e.g., Pika et al., 2003, 2005; Bonnie and de Waal, 2006; Liebal et al., 2006).^4^ Examples of group-specific gestures in wild chimpanzees include the above-mentioned auditory gestures of KNUCKLE-KNOCKING and LEAF-CLIPPING. Group-specific gestures are used in particular contexts and in some cases only by defined age groups or sexes. For instance, KNUCKLE-KNOCKING is found only in males in the North group of Taï National Park and used only in the mating context (Boesch, 2012a,b).

We now turn to potential candidates for basic symbolic signal use in natural great ape communication, concentrating on chimpanzee gestures. We present them here to inspire future research and to help illustrate the theoretical framework in the section “Possible pathways to basic symbolic communication.” Note that our suggestions are not based on large data sets but on the observations of long-term field researchers who observed these behaviors during their targeted data collection. There are mentions in the literature, but no systematic accounts, except for LEAF-CLIPPING (Nishida, 1980). Systematic research is needed to confirm the symbolic nature of these candidates.

Potential candidates for an arbitrary and conventionalized form-meaning linkage can, in our opinion, be observed in several group-specific gestures described for three neighboring chimpanzee groups in Taї National Park (Côte d’Ivoire): the North group, the East group, and the South group. For a map and more detailed information, see the section “NEST-BUILDING: a case study.” The use of gestures differs significantly from group to group (see Table 1). A male chimpanzee in the Taï South group may bend together a few branches or saplings when he wants to mate with a female, while in the North group, just a few kilometers away, a male would knuckle-knock for the same purpose (e.g., Boesch, 2003, 2012a). And a young chimpanzee of the East group or the South group builds a nest to invite peers to play, while in the North group holding a leaf in the mouth would be the appropriate signal (Boesch, 2012a; Luncz and Boesch, 2015). For an overview, see Table 1.

**Table 1:** Group-specific gestures in the three Taï groups.

Gesture	Form	Meaning	North group	South group	East group
NEST-BUILDING	Bending together (a few) branches or saplings	Invitation to play	−	+	+
		Sexual solicitation	−	+	−
KNUCKLE-KNOCKING	Knocking knuckles on hard surface	Sexual solicitation	+	−	−
LEAF IN MOUTH	Holding a leaf in the mouth	Invitation to play	+	−	?

+ = present (observed once a week); − = absent; and? = limited observation time (based on Luncz and Boesch, 2015).

The microcosm of these three habituated groups comprises no more than a few square kilometers. It is characterized by ecological similarity (Luncz et al., 2012) and by genetic relatedness between groups, which is due to migrating females^5^ and extra-group paternity (Schubert et al., 2011). And yet, different group-specific gestures have evolved (Boesch, 2003, 2012a,b). Table 2 draws out the apparently arbitrary linkage between form and meaning in these gestures.

**Table 2:** Arbitrary relation between form and meaning in Taï group-specific gestures.

	North group	South group	East group	North group
Form	Knocking knuckles on hard surface	Bending together (a few) branches or saplings		Holding a leaf in the mouth
Meaning	Invitation to mate		Invitation to play	

Note first that in these group-specific gestures, different forms are used to express one and the same meaning in different groups. For example, the meaning “invitation to mate” is conveyed by the form of knuckle-knocking in the North group but by bending together branches in the South group. And second, the same form is used to express different meanings within one group or within different groups. For example, the form of bending together a few branches conveys the meaning “invitation to mate” in the South group but “invitation to play” in the East group and in the South group. Because these gestures are not species-specific but group-specific, the arbitrary form-meaning linkage cannot be genetically determined; rather it must be socially transmitted at the group level (Boesch, 1991, 2012a). A detailed case study of NEST-BUILDING is provided below. Because there are comparatively little data concerning the LEAF-IN-MOUTH gesture, we will not include it in further analysis.

Another possible candidate for conventionalized and arbitrary form-meaning linkage would be, in our view, the auditory gesture of LEAF-CLIPPING. This gesture consists of removing parts of leaves with the mouth or fingers, thereby causing a distinctive sound (as mentioned in the *Introduction*). Nishida (1980) thought it likely that the gesture emerged from the preparation of fishing rods from leaves to catch tree-living ants, a non-social behavior.

LEAF-CLIPPING can be found in several wild chimpanzee communities where it is used in different contexts (see Table 3 for an overview). Thus, in Mahale (Tanzania) both males and females LEAF-CLIP for sexual solicitation (Nishida, 1980). This also holds for Budongo (Uganda; Hobaiter and Byrne, 2014) and Ngogo (Uganda; Watts, 2008). In Bossou (Guinea), female chimpanzees LEAF-CLIP in varied contexts (Sugiyama, 1981). In Taï (Côte d’Ivoire), males of the South group use this auditory gesture in the context of displaying; remarkably, it reappeared during an alpha-male takeover, after a gap of 2 years (Kalan and Boesch, 2018).

**Table 3:** LEAF-CLIPPING in different chimpanzee communities.

Community	Sender	Context
Mahale (Tanzania)	Males and females	Mating
Budongo (Uganda)	Males and females	Mating
Ngogo (Uganda)	Males and females	Mating
Bossou (Guinea)	Females	Varied contexts
Taï (Côte d’Ivoire) South group	Males	Displaying

Note that in the case of Bossou the gesture is used in varied contexts. The recipients need to discern the meaning from the accompanying behaviors. The gesture there seems to serve as a general attention getter that is conventionalized only in the sense that it is used by females exclusively. It draws the attention to the sender without in itself conveying context-specific meaning. Therefore, in this case, it cannot be considered as a potential candidate for symbolic signal use.

Contrary to that, LEAF-CLIPPING (except for Bossou) and KNUCKLE-KNOCKING do not seem to serve merely as general attention getters to direct the recipient’s attention to the sender. Rather, in addition to the attention getting component that (one could argue) is inherent to all auditory gestures, LEAF-CLIPPING and KNUCKLE-KNOCKING in themselves appear to convey information about the specific context/meaning in the respective groups. This group-specific meaning (e.g., sexual solicitation) would make further context-specific signals or clues superfluous.

Thus, these gestures would go beyond the characterization of *attention getters* given by Tomasello and Call (2007, 2019), according to which *attention getters* direct the recipient’s attention to the signaler; the recipient then has to discern the intended meaning from the accompanying behavior. We take this up in the section “Semantic shifts: a new perspective on the semantics of attention getters.”

Furthermore, LEAF-CLIPPING and KNUCKLE-KNOCKING do not seem to be learned individually but socially. While it cannot be excluded that, e.g., KNUCKLE-KNOCKING happens to be discovered and used by an individual to draw the general attention of conspecifics to him/herself, reports by fieldworkers confirm that in the Taï North group KNUCKLE-KNOCKING is used exclusively by young adult males, only for sexual solicitation, and has been observed across generations (e.g., Boesch, 2003, 2012a; Luncz and Boesch, 2015).

The form thus seems to be linked arbitrarily to one group-specific meaning. This linkage then would be conventionalized within the community and used and understood accordingly, even without further signals.

Boesch (2003) reports that young males in the North group use KNUCKLE-KNOCKING discreetly and repeatedly to attract females, who respond by presenting sexually. There are even instances when a different female presents to the sender although he was not looking in her direction. And significantly, sexually immature females may sexually present to the sender. That is to say, the meaning of the gesture is clearly understood by itself and this is not dependent on the sexual state of the recipient nor on the visual orientation of the sender.

In summary, we have proposed several candidates for learned arbitrary form-meaning linkage that appear to exhibit the criteria of *arbitrariness* and *conventionalization*. These signals thus, in our view, could be considered as potential candidates for a basic form of symbolic communication. Systematic field research is needed to confirm this view.

In the following section, we discuss possible pathways for the emergence of basic symbolic signal use in wild chimpanzee gestural communication. We propose that conventionalized arbitrary gestures can arise ontogenetically by borrowing the form of an existing gesture or the form of a non-social behavior that acquires communicative meaning. In both cases, the resulting (group-specific) gesture is used in a different context from that of the underlying gesture or non-social behavior, thus resulting in an arbitrary form-meaning linkage.

In the section “NEST-BUILDING: a case study,” we illustrate how a basic symbolic signal could emerge and operate. The visual gesture of NEST-BUILDING in chimpanzee natural gestural communication has not been described in detail in the literature so far. It can be observed in two chimpanzee groups in Taï National Park (Côte d’Ivoire). The gesture consists of bending together a few branches or saplings, and the possible contexts are mating and/or playing, depending on the group that uses it.

## Possible Pathways to Basic Symbolic Communication

We now explore how an arbitrary form-meaning linkage in the natural gestural communication of chimpanzees could come about, and how it could be conventionalized at the group level. First, we propose two routes for the emergence of arbitrary gestures within an ontogenetic time frame. We do so from a linguistic perspective.

### Two Routes to Arbitrariness

#### Emergence of Semanticity: A New Perspective on Non-social Behaviors in Gestural Ontogeny

We suggest that learned arbitrary gestures can emerge from *non-social behaviors* that acquire communicative functions. As described in the section “NEST-BUILDING: a case study,” the way that the “play nests” and the “mating nests” are built suggests that PLAY-NEST BUILDING and MATING-NEST BUILDING are based on the non-social behavior of nest building for resting. By non-social behaviors, we mean functional behaviors that are displayed outside social interactions and without a communicative purpose. That is, a non-social behavior (nest building for resting) may have developed into a social behavior and – by acquiring communicative meaning – passed to the gestural level. Note that the resulting gestures would exhibit an arbitrary form-meaning linkage, because the gestures are used in different contexts (playing/mating) from that of the underlying non-social behavior (resting). The same seems to hold for LEAF-CLIPPING, as summarized in Table 4 and described in the section “Criteria for basic symbolic signal use – and some potential candidates.”

**Table 4:** Group-specific gestures potentially based on non-social behaviors.

Gesture	Underlying non-social behavior	Old context	New context
NEST-BUILDING	Building nests	Resting	Mating/playing
LEAF-CLIPPING	Preparing leaf mid-ribs	Foraging	Varied (see Table 3)

This development needs to be distinguished from *phylogenetic ritualization* (Darwin, 1872; van Hooff, 1972, 2012; Krebs and Dawkins, 1984), where the form of non-social behaviors can be “borrowed” to serve a communicative function (*principle of derived activities*, Tinbergen, 1952). Over evolutionary time, *phylogenetic ritualization* results in species-specific gestures (gestural phylogeny). An example could be the dominance signal of MOUNTING in monkeys that may have evolved from mating behavior (Liebal and Call, 2012).

In contrast to this, we propose the emergence of new gestures from non-social behaviors within a much shorter time frame (gestural ontogeny). We call this development *emergence of semanticity*; here *semanticity* denotes the meaningfulness of communicative signals, one of the universal design features of human language as identified by Hockett (1960). This means that every communicative signal consists of a form and an associated meaning (de Saussure, 1916). The resulting gestures would be shared not at the species level (as in *phylogenetic ritualization*) but at the group level.

Theories of great ape gestural ontogeny so far heavily concentrate on the ritualization of social behaviors into gestures in social interaction (for instance, *ontogenetic ritualization* as proposed by Tomasello (1996) that results in *intention movements*, see below). Non-social behaviors are under-represented in these approaches. We now turn to the mechanism which may underlie this intriguing phenomenon.

**Figure 1:**
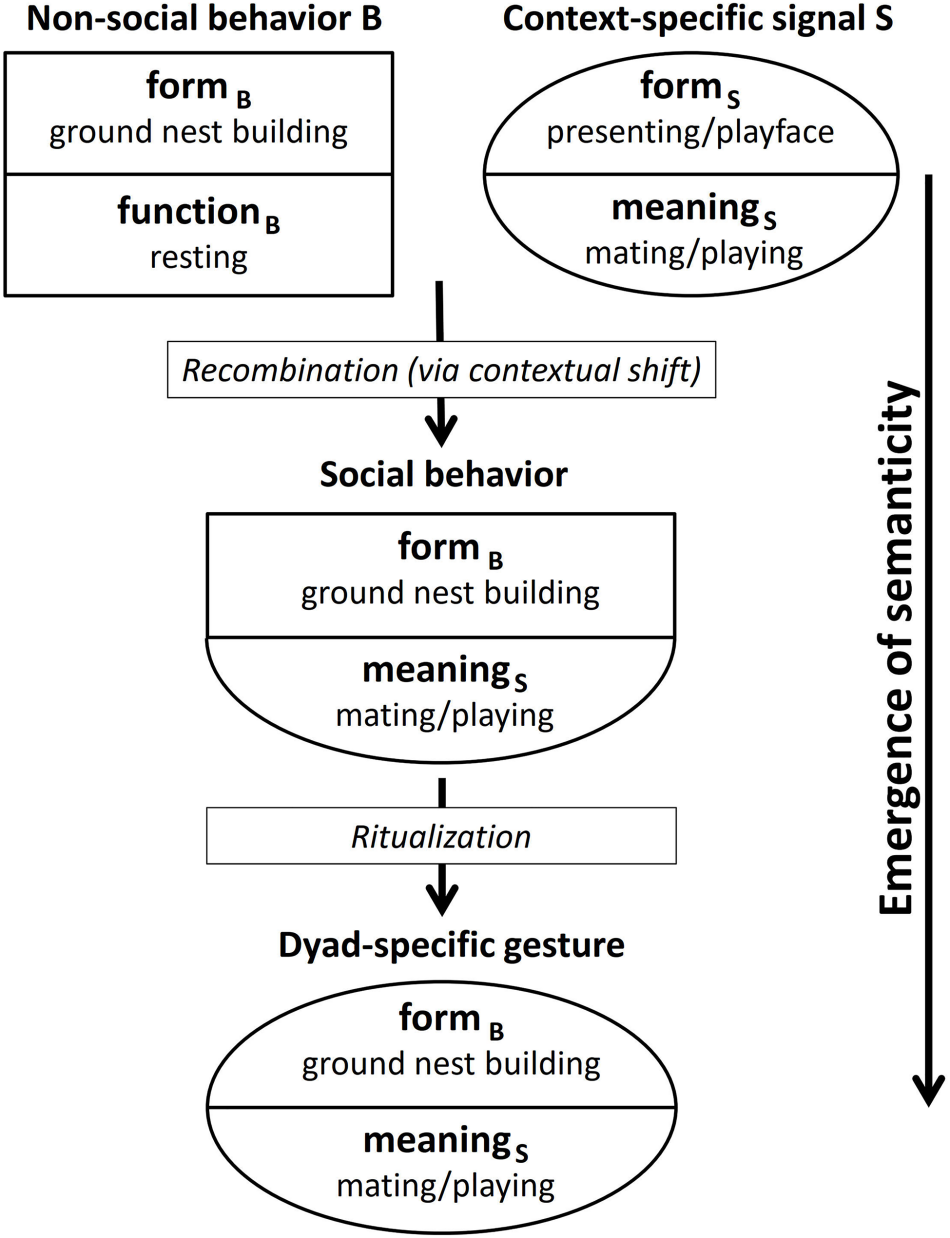
Emergence of a dyad-specific gesture from a non-social behavior.

The *emergence of semanticity*, as shown in Figure 1, comprises, in a first step, the recombination of the form of a non-social behavior B (form_B_: e.g., nest-building) with the meaning of a context-specific signal S (meaning_S_: e.g., sexual solicitation/play invitation) on the sender’s side. These context-specific signals carry a message about a communicative interaction. For chimpanzees, for instance, in the play context this might be a play face and/or play gait (van Hooff, 2012; see Wilson, 1975 for other species). In the mating context, the presenting of an erect penis defines the communicative context and thus determines the meaning of any other signals used in combination by the male, for instance, BRANCH SHAKING and STOMPING.^6^

Thus, one could argue that the meaning of these context-stressing signals is imposed onto all signals sent at the time. However, signaling normally does not happen in a vacuum. It is not separate from other processes going on simultaneously in the environment of the sender and the recipient. Therefore, non-social behaviors exhibited by the sender that have nothing to do with the current communicative context may be drawn into the contextual field and become “colored” with context-specific meaning. Of course, it is partly a matter of chance which of the myriad of potential behaviors happening in parallel with communicative interactions (or temporarily close enough to them) are associated with the communicative context, so that the behavioral form is recombined with the meaning of the communicative signals being used.

At first, the sender may include the behavior just “because,” that is, because it happened to be part of a successful interaction, even if it was not meant to be communicative. Repeated successful use of the behavior in connection with other context-specific signals may then result in a recombination of form_B_ (provided by the nest-building behavior of the sender) and meaning_S_ (provided by one or more context-specific signals) on the sender’s side.

The new form_B_-meaning_S_ combination would result from a contextual shift. In the cases of PLAY-NEST BUILDING and MATING-NEST BUILDING, this means a shift from the context of resting to the context of playing/mating. In the case of LEAF-CLIPPING, the context of the underlying non-social behavior (fishing for ants) is foraging, while the resulting gesture is used, for instance, in the mating context, as illustrated in Table 4. The form_B_-meaning_S_ combination can be regarded as a social behavior (that is, socially directed by the sender toward a recipient) although in its form being based on a non-social behavior.

In a second step, this social behavior is then ritualized into a dyad-specific gesture. By dyad-specific gesture, we mean a gesture that arises within a particular dyad and is used by one or by both individuals. This ritualization is a social process that takes place within an ontogenetic time frame. During the ritualization process, the behavior may get abbreviated/truncated as proposed for *intention movements* that are based on social behaviors.

The ritualization process in the *emergence of semanticity* differs from *ontogenetic ritualization sensu* Tomasello (1996). *Ontogenetic ritualization* results in *intention movements* whose forms still represent part of the underlying (social) behaviors. Thus, the form and the meaning of gestures resulting from *ontogenetic ritualization* are connected logically and not arbitrarily. The *emergence of semanticity*, in contrast, results in gestures that do not exhibit a logical but rather an arbitrary connection between the form and the meaning of the gestures. As explained above, the reason for this phenomenon lies in the fact that the resulting gesture is used in a different context from that of the underlying non-social behavior (contextual shift).

In all examples listed above, a non-social behavior may have acquired communicative meaning. The resulting gestures would disappear with the individuals that use them, or even at some point within the individuals’ lifetime. To survive, they need to be copied by other group members and thus develop into group-specific gestures. We analyze this next step in the following section “Conventionalization.”

In summary, we suggest that ontogenetically arising chimpanzee gestures can be based on non-social behaviors. In this process, the form of non-social behaviors is recombined with the meaning of co-occurring context-specific signals, resulting in arbitrary form-meaning linkage. Note that in the case of NEST-BUILDING, the form of the underlying non-social behavior would have been truncated. One could thus argue that it resulted in an *intention movement* – but with arbitrary form-meaning linkage. The mechanism of *emergence of semanticity* hence would take further the concept of *intention movement* defined by Tomasello and Call (2007, 2019) by giving it an arbitrary form-meaning linkage.

#### Semantic Shifts: A New Perspective on the Semantics of Attention Getters

**Figure 2:**
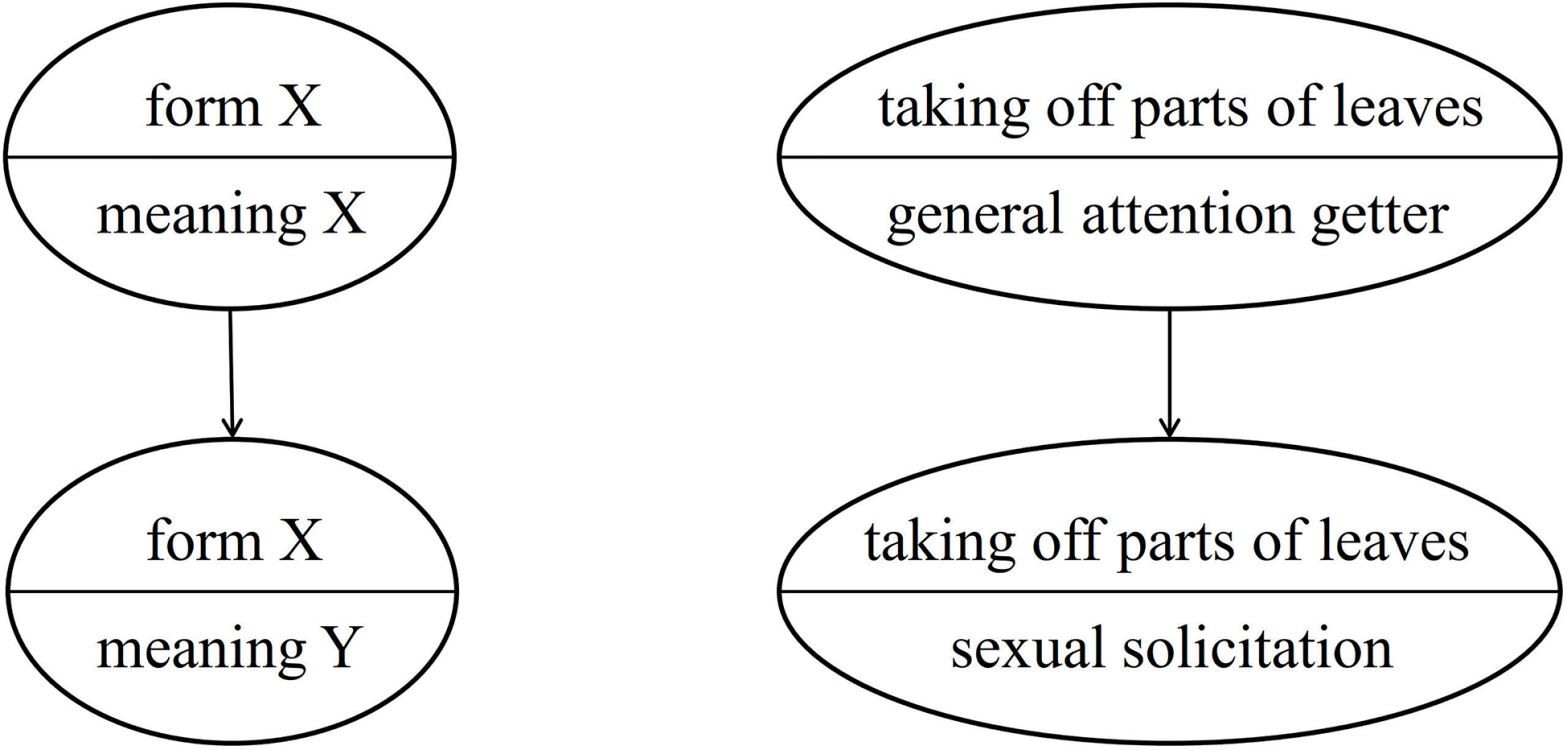
Group-specific semantic shift.

As Cissewski and Boesch (2016) have proposed, great apes may use *semantic shifts* to express new meanings without creating new forms. That is, within a community the meaning of an existing gesture would change without the form of the gesture being modified. This mechanism may underlie the group-specific usage of auditory gestures such as LEAF-CLIPPING (changing from a general attention-getter to a context-specific gesture). Importantly, the original meaning (general attention getter) of the gesture disappears, see Figure 2.

The reason for an existing group-specific gesture undergoing a semantic shift may be a gap in the communicative repertoire that needs to be filled, for instance, in communication under time pressure or in environments with restricted visibility. Using the gesture with the new (more specific) meaning under these circumstances would provide the sender and the recipient with adaptive benefits (for an example, see the section “Conventionalization”). A communicative gap thus can act like a vacuum that pulls existing elements into a different position in the communicative repertoire.^7^

Cissewski and Boesch (2016) argued that this phenomenon can be observed especially in auditory gestures (for instance, LEAF-CLIPPING and KNUCKLE-KNOCKING), as in this gestural modality form and meaning are less closely linked than in visual or tactile gestures that may result from *ontogenetic ritualization*. However, if a gesture of the visual or the tactile modality already exhibits arbitrary form-meaning linkage, then a semantic shift might become more feasible/likely. This could have been the case for NEST-BUILDING (see the case study below).

##### Excursus

**Figure 3:**
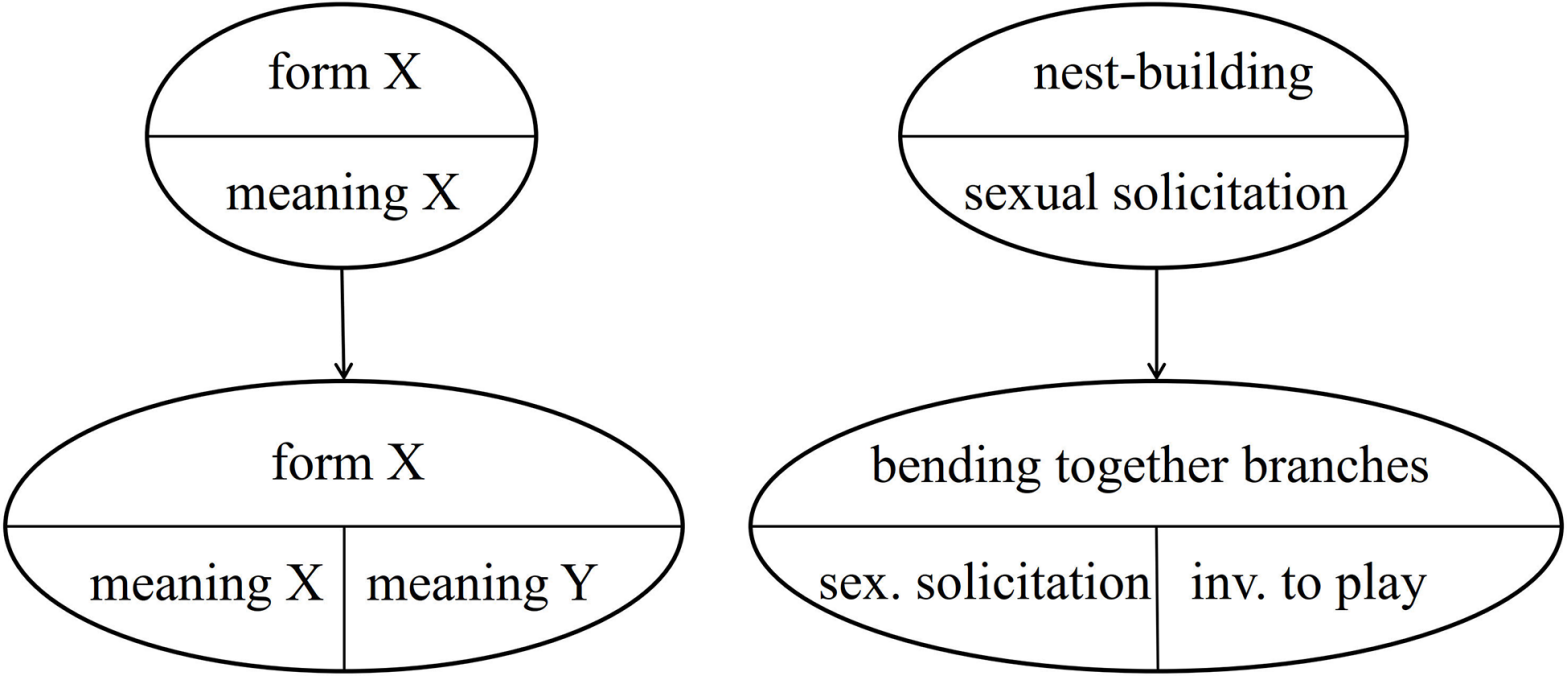
Partial group-specific semantic shift.

The case of NEST-BUILDING is even more interesting, because in the South group the gesture is used in two different contexts (see Table 5), each specific to an age group. So far, we assumed that the ground-nest gestures used in the play context and in the mating context in the Taï South group emerged independently. However, it is also possible that one is based on the other. This would mean that either the ground-nest gesture used by adults in the mating context was copied by infants for the play context, or vice versa. Thus, the meaning of an existing group-specific gesture would have been modified – within an age-group. However, the original meaning is kept in the adults (or the infants, respectively), see Figure 3 below.^8^ This would mean that the semantic shift is age-group specific and that it is only partial. Both meanings exist in parallel.^9^

**Table 5:** Properties and distribution of NEST-BUILDING.

Gesture	Form	Meaning	Sender	North group	South group	East group
PLAY-NEST BUILDING	Bending together a few branches or saplings	Invitation to play	Juveniles and adolescents of both sexes	−	+	+
MATING-NEST BUILDING		Sexual solicitation	Adult males	−	+	−

Note that the mechanism of group-specific semantic shifts takes further the concept of attention getters defined by Tomasello and Call (2007, 2019), by adding context-specific meaning. The gesture thus does not only draw the recipient’s attention to the sender, but at the same time also includes the information as to why the attention is sought (e.g., sexual solicitation), without additional behavioral cues. The resulting gesture is not an intention movement either, but a gesture with arbitrary form-meaning linkage. Systematic field research is needed to establish that context-specific meaning is communicated by these gestures themselves, without any other context-specific signals being present (or discernable to the recipient).

In summary, we suggest that in some cases it is possible that attention getters undergo further development, by acquiring context-specific meaning. This would result in an arbitrary form-meaning linkage that is conventionalized first at the dyadic level and then at the group level (as discussed in the following section).

### Conventionalization

The second criterion for basic symbolic signal use is *conventionalization*. True symbols cannot be innate but must be learned, in order to be shared by the members of the group. *Phylogenetic ritualization* (Darwin, 1872; van Hooff, 1972, 2012; Krebs and Dawkins, 1984), as described above in the section on the *emergence of semanticity*, results in species-specific gestures and thus would not provide an explanation for the existence of group-specific gestures. *Ontogenetic ritualization* (e.g., Tomasello, 1996), as also described in the section on the *emergence of semanticity*, results in gestures shared within dyads; it may take place in parallel in different dyads of a group, based on the same functional actions. The hypothesis of *ontogenetic ritualization* has been challenged, for instance by Genty et al. (2009) and Hobaiter and Byrne (2011). However, there is recent evidence in support of *ontogenetic ritualization* in bonobos (Halina et al., 2013). For *ontogenetic ritualization* to result in stable group-specific gestures across generations, gestures would need to be ritualized over and over again (see Genty et al., 2009). Cartmill and Hobaiter (2019) thus suggest experiments for testing whether gestures resulting from *ontogenetic ritualization* would be transferable to a *new* partner, that is, outside the original dyad. Byrne et al. (2017) propose the innateness of the majority of great ape gestures; this includes group-specific gesture as being the result of different developmental environments.

Another possibility for the rise of group-specific gestures would be social transmission. Unfortunately, little is known about the precise mechanisms of the spreading of new gestures within groups of great apes (for a review, see Liebal et al., 2019). In view of the richness of social learning mechanisms (e.g., Hoppitt and Laland, 2008, 2013), the precise process of the conventionalization of a gesture at the group level cannot be determined retrospectively.

One could argue that the mechanism underlying the spread of NEST-BUILDING, LEAF-CLIPPING, and other group-specific gestures is observational learning, given the evidence of observational learning in captive apes (e.g., Whiten et al., 2004). Moreover, as pointed out in footnote 5, migrating females normally adjust to the cultural givens of their new group (Luncz et al., 2012; Luncz and Boesch, 2014). This includes gestures. We suggest that observational learning is a worthwhile hypothesis for field researchers to investigate further.

In practice, in the mating context, seeing the demonstrator being successful with a mating partner, should be sufficient motivation for the observer to learn the gesture. There are two variants here. The observer male might adopt the behavior specifically to attract the same female who reacted to it with the demonstrator, and this could be successful. Or the male might adopt the behavior with a different female, who has not seen it previously in this context. However, this is still a strategy which may work, since context specific-signals accompanying the new gesture would define the context. Through usage in repeated interaction, the gesture would be associated with mating in an increasing number of individuals and eventually become group-specific. In the play context, if the demonstrator successfully attracts playmates, the observer is likely to be motivated to adopt this behavior.

Note that we are not dealing with *response facilitation*, that is, NEST-BUILDING, LEAF-CLIPPING, and KNUCKLE-KNOCKING do not need the presence of a demonstrator to be displayed in the appropriate context in every-day social interaction. And we are not dealing with *program-level imitation*, because there is no novel organization of several preexisting components happening. Further note that, intriguingly, in gestures resulting from *the emergence of semanticity* or from *semantic shifts*, the form of the gesture would already have been part of the behavioral repertoire, either belonging to a non-social behavior or to a gesture. The form thus would not need to be learned.

Why then would group members adopt new gestures? As we have already mentioned, gestures may spread within a group because they provide the sender and/or recipient with adaptive benefits under specific social and ecological circumstances. Thus, to stay with the visual gesture of NEST-BUILDING, the building of mating nests enlarges the number of gestures available for sexual solicitation and thus can increase the level of persistence. Using and understanding the gesture might therefore provide an adaptive benefit in the mating context.

As proposed by Cissewski and Boesch (2016), in habitats where visibility is restricted, group-specific *semantic shifts* in auditory gestures may result in more effective communication. For instance, when signaling under time pressure, the rapid communication of meaning *via* the auditory modality provides an adaptive benefit for the signaler and/or the recipient. This could be relevant in the mating context, when mating access for males is mainly controlled by dominants while female choice is limited by male coercion. Conventionalized inconspicuous KNUCKLE-KNOCKING lets subordinate males gain mating opportunities and females gain female choice. These are strong adaptive reasons for conventionalization. Moreover, according to anecdotal evidence from Taï (Deschner, personal communication), the audience moves away from the sender when hearing LEAF-CLIPPING, because they expect an upcoming display. This reduces the risk of confrontation for the audience – and the sender.

Crucially, the effect of adaptive benefits is not strong enough to ensure that all communities with similar material and social environments converge on the same group-specific gestures. Great apes and other nonhuman primates live in complex material and social environments (e.g., Milton, 1981; Russon and Begun, 2004; Cheney and Seyfarth, 2007). The forest is no laboratory with controlled conditions. It is complex with many factors acting and interacting.

Therefore, in addition to gaining/providing adaptive benefits, we should allow for the possibility that new gestures or other behaviors may get copied without an obvious adaptive benefit, but simply because this is “how it is done.” This might be the case for the generalization of PLAY-NEST BUILDING in the Taï South and East groups. Comparable scenarios have already been reported in the literature. Thus, van Leeuwen et al. (2014) report on the spontaneously emerged tradition of “grass-in-ear behavior” in one chimpanzee group of the Chimfunshi Wildlife Orphanage (Zambia). A female repeatedly put a piece of grass in her ear and left it there. Soon, other group members copied this behavior which does not have any apparent adaptive value. Another instance would be the copying of the individual-specific manner of back scratching performed by a chimpanzee with snare-damaged hands in the Ngogo community of Kibale National Park (Uganda); the copying by group members without the injury did not seem to be adaptive (Hobaiter and Byrne, 2010).

In such cases, the copying of the new behavior seems to result from a general predisposition to copy. This predisposition might have been selected for, because in itself it provides an adaptive benefit because it allows for useful behaviors to be acquired. However, the specific behaviors copied may not in every case provide an adaptive benefit. Thus, we propose that the emergence of a particular group-specific behavior, including gestures, does not need to be driven directly by adaptive benefits.

## Nest-Building: A Case Study

**Figure 4:**
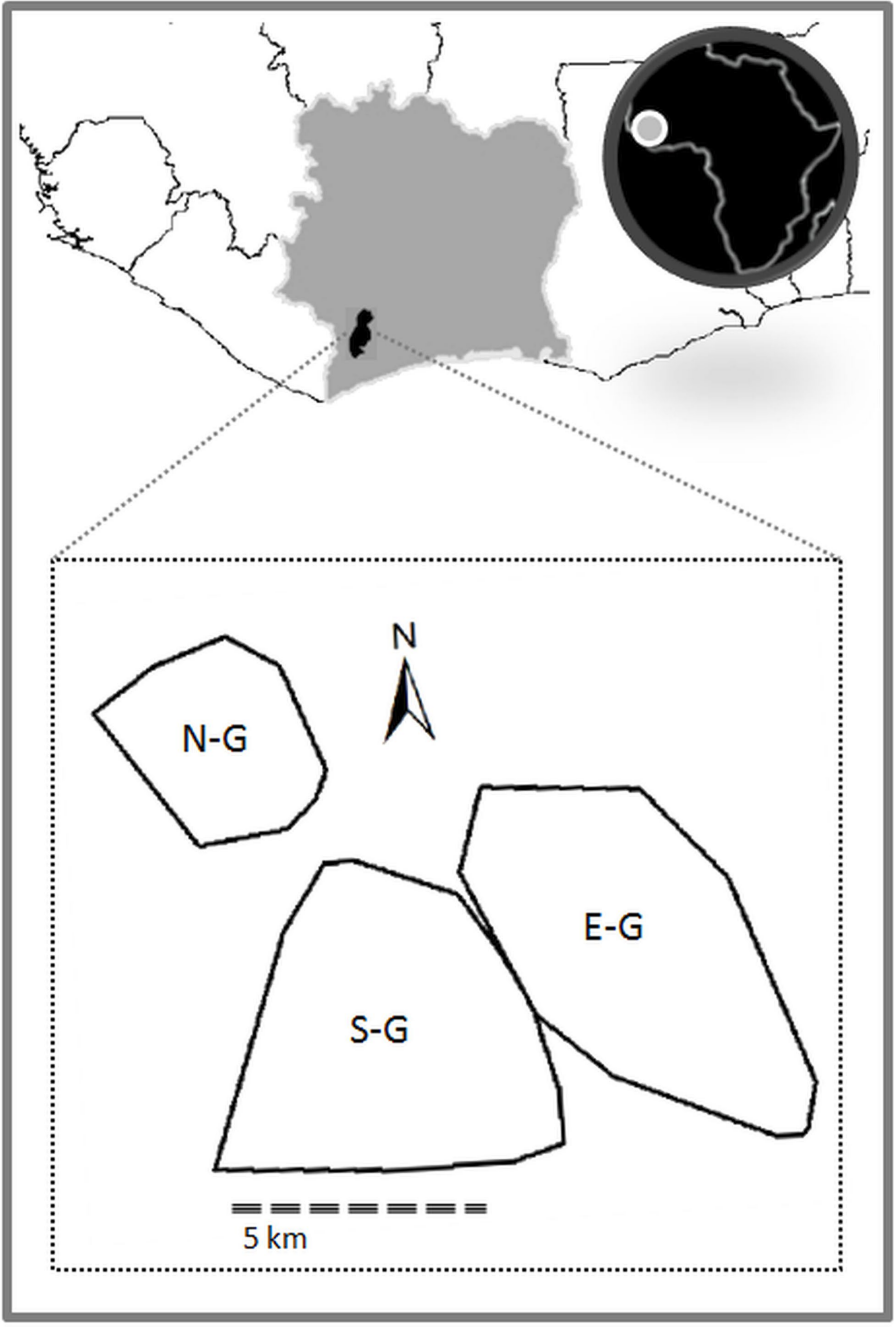
The three habituated chimpanzee communities in Taï National Park, Côte d’Ivoire (N-G = North group; S-G = South group; and E-G = East group). Polygons indicate the home ranges of the chimpanzee groups at time of observation (2007–2009).

We now discuss in detail one of the potential candidates for basic symbolic signal use: NEST-BUILDING. This intriguing phenomenon is found in two of the three study groups in Taï National Park (see Figure 4) and has not been observed in other wild chimpanzee communities. So far it has not been studied systematically nor been described in detail in the relevant literature (for mentions in the literature, see, for instance, Boesch, 2012a and Luncz and Boesch, 2015). We therefore strongly encourage fieldworkers to undertake systematic data collection and analysis to test our hypotheses.

These groups are fully habituated to the presence of humans and have been continuously observed since 1983 (Boesch and Boesch-Achermann, 2000). They engage in frequent violent intergroup encounters (Samuni et al., 2017), which do not allow them the opportunity to observe the daily behavior of members of the other groups (Boesch et al., 2008).

The gesture of NEST-BUILDING consists of bending together a few branches or tree saplings. Note that NEST-BUILDING is not to be confused with the purely functional and non-social behavior of nest-building for resting, although it is possible that the gesture borrowed its form from this non-social behavior (as proposed for the *emergence of semanticity*). Further note that the very simple constructions resulting from the gesture of NEST-BUILDING do not resemble real nests like those constructed for resting. This is clear in the video material provided for illustration. To emphasize this important distinction and to avoid misunderstandings, we first briefly describe the non-social behavior of nest building for resting and then we discuss in detail the actual gesture of NEST-BUILDING.

### Nest-Building for Resting: A Non-social Behavior

Wild chimpanzees build nests, for sleeping during the night, and for resting during the day (for reviews, see Fruth and Hohmann, 1996 as well as Hicks, 2010). Day nests for resting are normally simpler than the more elaborate night nests. The chimpanzee communities of the Taї National Park follow this pattern, and day nests are commonly constructed for resting (Boesch, 1995). Although simpler than the night nests, these day nests are built by bending branches and/or saplings together, interweaving them and adding torn twigs and branches. Day nests are usually built on the ground, though sometimes also in the trees. The resting nests are normally used by one chimpanzee at a time, unless a mother has a dependent offspring. They are not used as sites for play or mating.

Video 1 illustrates the building of a day nest in a tree for resting (note that the individual briefly interrupts the building process in order to retrieve food that has been accidentally dropped).

Video 1: https://share.eva.mpg.de/index.php/s/noTsjAJCmrRs6cm(Copyright: Liran Samuni, Taï Chimpanzee Project).

Again we emphasize that the building of day nests in the resting context is a non-social behavior. That is, it is a functional behavior that is not directed at other individuals and thus lacks communicative intent. It takes place without monitoring the attention of others, without waiting for a response and without receiving a response from other individuals.

### Nest-Building in Communicative Interaction

As we have seen, chimpanzees build nests for resting, which is a merely functional and non-social behavior. In addition, in the Taï East group and the Taï South group they may exhibit the activity of bending together vegetation with communicative intent.

The resulting gesture is called NEST-BUILDING, because the form of the gesture resembles the motorics of the act of nest-building for resting. However, the gesture does not result in a full nest but in something much simpler (see Videos 2 and 3). Note also that it is not the resulting “play nests” and “mating nests” themselves but the actual process of bending together the small number of branches/saplings that has acquired communicative meaning. Similarly, the auditory gesture LEAF-CLIPPING, described above, consists precisely in the act of taking off parts of leaves and not in the bare mid-ribs that result.

In the Taï South group, NEST-BUILDING occurs in the mating context and in the play context (MATING-NEST BUILDING and PLAY-NEST BUILDING), and in the Taï East group in occurs in the play context (PLAY-NEST BUILDING; Boesch, 2012a; Luncz and Boesch, 2015). See Table 5 for an overview. A detailed description is provided in the following.

Note that the building of nests does not serve any function in the play or in the mating contexts in the Taï South and East groups (e.g., Luncz, personal observation) outside the communicative interactions described in the following. This is important with regard to the arbitrariness of the form-meaning linkage of these gestures. Outside communicative interactions, the form (bending together a few branches or saplings) is not linked to the mating or the playing context. Thus, the nest is not linked in form to the response of the recipient or to the subsequent behavior of the signaler. The form-meaning linkage in MATING-NEST BUILDING and PLAY-NEST BUILDING would thus be truly arbitrary.

#### PLAY-NEST BUILDING

In order to initiate play, juvenile and adolescent chimpanzees in the East group and the South group are frequently seen bending a few surrounding saplings or branches together (e.g., Boesch, 2012a; Luncz and Boesch, 2015; e.g., Crockford et al., personal communications). Even though this behavior is observed frequently by different field researchers, there are as yet no systematic data on the use of PLAY-NEST BUILDING.

However, from September 2007 to November 2009 data were collected opportunistically by Luncz during focal follows of adult individuals, resulting in 44 independent observations of PLAY-NEST BUILDING in the East and the South groups^10^; there were, in addition, many more instances of PLAY-NEST BUILDING which were not recorded, because the researcher was focusing on adult individuals. The gesture was observed in juveniles and adolescents, both male and female, aged from 2 up to 12 years to initiate play. Both sexes responded to such play invitations. PLAY-NEST BUILDING was most frequently observed during the resting times of adult group members, a period when offspring play time is increased. Unlike resting nests, these “play nests” do normally not leave any physical evidence after play as the saplings generally regain their original structure. The saplings usually only get bent and not broken.

The sender bends together a few branches or saplings in proximity to a potential play partner (at a clear visual distance of approximately 1–5 m), taking into account the recipient’s attentional state. The builder may exhibit visual checking toward the potential recipient. During or immediately after construction, which in general takes only a few seconds, the selected play partner may join the builder by interrupting him/her and play begins. Hence, the sender receives a voluntary response, that is, the potential recipient is not pulled into the nest. The building is mechanically ineffective toward the recipient. If the play partner does not react to the invitation during construction or immediately after, the builder usually sits down on the bent-over branches and looks at the potential play partner, thus exhibiting response waiting. If still nothing happens, a second round may be started or a different strategy be applied (e.g., pulling the other’s leg). Note that due to the usually almost immediate reaction of the play partner, markers of intentionality like persistence or elaboration on the sender’s side (e.g., by adding a second round) are hardly ever needed. Importantly, the bent-over branches clearly do not serve the purpose of resting as young chimpanzees were never observed to lie down on them. Thus, the construction resulting from PLAY-NEST BUILDING is not perceived as a nest and is not occupied by both.

Video 2 shows an example of PLAY-NEST BUILDING. Two infants play in an old resting nest in a tree. One stops and leaves the nest. The other reacts with PLAY-NEST BUILDING, and the first individual accepts the invitation and play is resumed.

Video 2: https://share.eva.mpg.de/index.php/s/tRWWbbLHAAzYcjR(Copyright: Liran Samuni, Taï Chimpanzee Project).

The reduced building process emphasizes the communicative intent, being clearly distinguishable from the original underlying behavior (nest building for resting, as shown in Video 1), especially given that it is carried out while the adult individuals are resting.

Importantly, PLAY-NEST BUILDING is interpreted as play invitation also in the absence of play-context specific signals like the play face (Luncz, personal observation; Christophe Boesch, personal communication). The gesture thus does not need any pragmatic support and serves as play invitation in its own right. PLAY-NEST BUILDING as a gesture for play invitation thus seems to be truly referential. Of course, it needs systematic data collection, ideally through video recordings, to provide firmer empirical evidence for the independent use of the gesture.

The points listed above clearly differentiate PLAY-NEST BUILDING from the building of nests in the resting context; the latter takes place without monitoring the attention of other individuals, does not include response waiting, and is finished without receiving a response from other individuals. As detailed above, nest-building serves no function in the play context outside the communicative interactions described here. This suggests that the form of the gesture (bending a few saplings or branches together) and its meaning (play invitation) are linked arbitrarily; that is, there would be no logical connection between the two. This arbitrary linkage would be shared and thus conventionalized at the group level. Note that PLAY-NEST BUILDING has been observed for about two decades, showing its sustained use over generations and in this way providing evidence for acquisition *via* social learning. Thus, PLAY-NEST BUILDING, in our view, can be regarded as a potential candidate for investigating symbolic signal use.

#### MATING-NEST BUILDING

**Figure 5:**
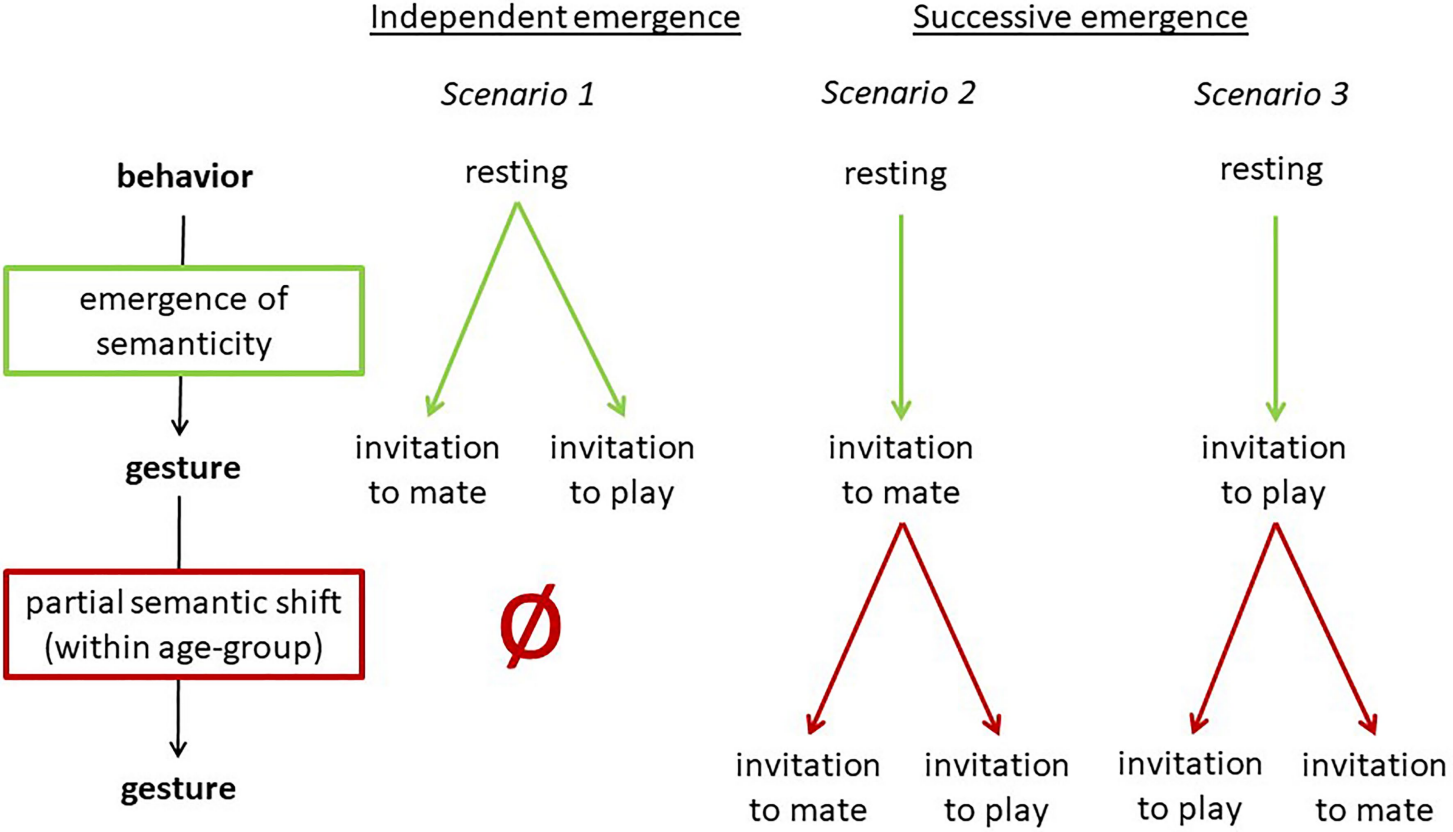
Three scenarios for the emergence of PLAY-NEST BUILDING and MATING-NEST BUILDING in the Taï South group.

In addition to being used to initiate play by juveniles and adolescents, in the Taï South group (but not in the East group), the bending together of a few branches is used communicatively by adult males for sexual solicitation (e.g., Boesch, 2009, 2012b). Thus, the gesture in the South group is used in two contexts (each by one age-group) with two different meanings (invitation to play and sexual solicitation, see Table 5). It is unclear whether they evolved independently or whether one is based on the other. The latter would indicate a semantic shift as defined by Cissewski and Boesch (2016) and as described in the above section on semantic shifts, in this case limited to an age group. This would entail that the meaning of the gesture changed when the gesture was adopted by a different age group. This is visualized in Figure 5.

There are no systematic data on the use of MATING-NEST BUILDING. But as in the case of PLAY-NEST BUILDING, fieldworkers agree that the bent-over branches or saplings do not serve the original purpose of resting and that they exhibit communicative intent (e.g., Luncz, personal observation; Boesch, personal communication; Boesch, 2012a). MATING-NEST BUILDING is observed less frequently than PLAY-NEST BUILDING, because mating occurs less frequently than play and because MATING-NEST BUILDING (unlike PLAY-NEST BUILDING) is used only by male individuals.

MATING-NEST BUILDING consists of the quick bending together of a small number of branches or saplings, in close proximity to the female recipient and thus clearly audible and at least partly visible to her; it is done taking into account the potential mating partner’s attentional state. The form of the resulting nests is usually simpler than that of day nests for resting, but they can get more elaborate if the recipient does not react quickly. The sender (the male) does not lie down on the branches after construction, he visually checks the attention of the recipient, and he exhibits response waiting. The sender receives a voluntary response, that is, the potential recipient (the female) is not pulled toward the sender. MATING-NEST BUILDING is thus mechanically ineffective toward the recipient. Due to reluctance of the potential mating partner, persistence or elaboration on the sender’s side (e.g., by adding other context-specific signals) is often needed.

Thus, while PLAY-NEST BUILDING can often be observed as a “stand alone” gesture, MATING-NEST BUILDING typically occurs in connection with other context-specific signals like, for instance, the presenting of an erect penis. It also often becomes part of sequences of gestures with equivalent meaning (that is, sexual solicitation).

Video 3 shows a young chimpanzee male trying to convince a female (in the front with her back toward the camera) to mate with him. Due to her reluctance, the male uses a series of gestures, including MATING-NEST BUILDING at the beginning and, very rudimentary, in the middle of the sequence. The communicative intent of MATING-NEST BUILDING is nicely evident in the clip (e.g., monitoring the attention of the recipient, awaiting recipient’s response).

Video 3: https://share.eva.mpg.de/index.php/s/AKH27jnrbLFy3Kp(Copyright: Liran Samuni, Taï Chimpanzee Project).

It is especially younger males who use this gesture (Luncz, personal observation), probably because more persuasion is necessary for an adult female to mate with them. The gesture here often seems to serve the purposes of persistence and elaboration, to persuade a female to accept the male’s invitation. MATING-NEST BUILDING thus enlarges the number of gestures available for sexual solicitation and provides an additional means of persuading a female (especially an older female) to accept the male’s invitation.^11^

In addition, MATING-NEST BUILDING is an inconspicuous means of signaling. In environments, where visibility is restricted and there is time pressure on signaling, it can be advantageous to have an inconspicuous signal that can attract the attention of a female situated within several meters, but not the attention of a dominant male further away. Moreover, the sender cannot be identified acoustically by distant group members. The lower-ranking males generally pay attention to not display the behavior in the vicinity of the alpha male so as to not be detected. Thus, by adopting the gesture, subordinate males may gain mating opportunities, and by reacting to it, females may gain choice of partners.

Crucially, as stated above, there is no logical connection between the building of proper nests and mating in the Taï South group. Real nests are not used for mating. Note further that the rudimentary construction that results from MATING-NEST BUILDING has no role in actual mating, because the attracted female approaches the sender and sexually presents outside the area of the construction. Thus, the form of the gesture (bending a few saplings or branches together) and its meaning (sexual solicitation) would be linked arbitrarily (without a logical connection between the two) and the linkage would be conventionalized at the group-level. Note that MATING-NEST BUILDING (like PLAY-NEST BUILDING) has been observed for about two decades, showing its sustained use over generations and in this way providing evidence for acquisition *via* social learning. The reduced form of the resulting constructions further emphasizes the communicative intent of MATING-NEST BUILDING by making it distinguishable from the original underlying behavior (nest-building for resting). Given the points made above as well as the fact that MATING-NEST BUILDING can be used on a par in sequences with other established gestures (BRANCH SHAKING, PRESENTING PENIS), we propose that MATING-NEST BUILDING can be considered as a potential candidate for symbolic signal use for sexual solicitation in the Taï South group. However, more observational data are needed to establish whether MATING-NEST BUILDING is truly referential, that is, whether it is reliably understood by itself as a gesture for sexual solicitation. In contrast, PLAY-NEST BUILDING is reliably understood without further cues.

In summary, given that PLAY-NEST BUILDING and MATING-NEST BUILDING are group-specific gestures and thus cannot be found in other groups across the species, nor on the sub-species level, we would assume emergence and social transmission within an ontogenetic time frame, instead of innateness. The apparent arbitrary linkage between the gesture’s form (bending together a small number of branches or saplings) and its meaning (play invitation/sexual solicitation) thus would be learned and in our opinion might constitute evidence for basic symbolic communication. However, systematic data collection is needed for the case to be conclusive.

### Three Scenarios for the Emergence of the Ground-Nest Gesture in the Taï South Group

Based on the two processes of *emergence of semanticity* and *partial semantic shifts*, in this case study, we now apply these processes to the emergence of PLAY-NEST BUILDING and MATING-NEST BUILDING in the South group. We distinguish between two types of emergence: Independent emergence of PLAY-NEST BUILDING and MATING-NEST BUILDING in the two different age groups (scenario 1), and successive emergence, with MATING-NEST BUILDING (or PLAY-NEST BUILDING, respectively) being used first and then undergoing a partial semantic shift (scenarios 2–3) as suggested above and illustrated in Figure 3. An overview of the scenarios is provided in Figure 5.

## Conclusion

Our aim is to contribute to our understanding of the developmental origins of great ape gestures and to inspire researchers studying wild primates (and other species) to systematically investigate group-specific gestures, and other learned communicative elements, against the background of potential symbolic signal use.

For identifying a basic form of symbolic signal use in great ape natural communication, we have provided a theoretical framework based on the key criteria of *arbitrariness* and *conventionalization*. The form-meaning linkage of a gesture thus would be *arbitrary* if there is no obvious logical or otherwise motivated connection between the form and the meaning. And the linkage would be *conventionalized* if the gesture is not innate but learned by the members of the respective groups.

For the rise of conventionalized arbitrary gestures, we have proposed two routes: *semantic shifts* (a change of meaning in an existing gesture) and the *emergence of semanticity* (the creation of new gestures on the basis of non-social behaviors). In both cases, the resulting gesture would exhibit an arbitrary linkage of form and meaning, because the form of the gesture was borrowed from a behavior outside the context in which the resulting gesture is used. This arbitrary linkage would be conventionalized at the group-level, resulting in a group-specific basic symbolic gesture.

Furthermore, we have suggested potential candidates for basic symbolism in chimpanzee natural gestural communication. These candidates seem to exhibit the key characteristics of symbolic signal use in our framework: an arbitrary and conventionalized form-meaning linkage. Compared with the symbolic capacities demonstrated by great apes in laboratory environments, these candidates suggest symbolic signal use in chimpanzee natural gestural communication to be rather limited both with regard to the number of possible candidates and with regard to the number of contexts. However, future systematic field research and analysis may reveal a richer picture both in number of examples and in their variety.

Our focus on the basic characteristics of symbolic signal use together with the suggestive data from the field shed new light on the existence, nature, and origin of chimpanzee symbolic gestural communication. By making the case for arbitrary and conventionalized signals to be accepted as a sufficient characteristic for the presence of basic symbolic signal use, we hope to widen the scientific perspective on symbolic communication across species boundaries and to contribute to a more complete assessment of the presence of symbolic gestures in our closest living relatives, the great apes.

## Data Availability Statement

The original contributions presented in the study are included in the article/supplementary material, further inquiries can be directed to the corresponding author.

## Author Contributions

JC wrote the first draft of this manuscript. All authors contributed to the article and approved the submitted version.

## Conflict of Interest

The authors declare that the research was conducted in the absence of any commercial or financial relationships that could be construed as a potential conflict of interest.

## Publisher’s Note

All claims expressed in this article are solely those of the authors and do not necessarily represent those of their affiliated organizations, or those of the publisher, the editors and the reviewers. Any product that may be evaluated in this article, or claim that may be made by its manufacturer, is not guaranteed or endorsed by the publisher.

## Footnotes

^1^The same is true of human language, where we have very broad and very narrow concepts. Furthermore, in human language the degree of overlap between the speaker’s intention and the hearer’s understanding also varies dramatically.

^2^From here on gestures are set in SMALL CAPITALS.

^3^Since the term “conventionalization” is used in linguistics to describe the spreading of a vocabulary item among members of a speech community, we adopt it here in our theoretical framework.

^4^Group-specific vocalizations in the wild have been reported for chimpanzees (Crockford et al., 2004) and for orangutans (Wich et al., 2012), as well as for chimpanzees in captivity (Watson et al., 2015).

^5^Chimpanzee females are the dispersing sex, which means that with the onset of puberty females leave their native group and integrate into a new (possibly neighboring) group. The migrating females normally adjust to the cultural givens of their new group (Luncz et al., 2012; Luncz and Boesch, 2014). In this way, the behavioral repertoires of the neighboring groups are preserved over time and cultural differences can be observed for several behaviors (Luncz et al., 2012). This includes the acquisition of group-specific gestures. This could mean that the acquisition of gestures is a flexible behavior throughout the lifetime.

^6^Note that BRANCH SHAKING and STOMPING under these circumstances only initially serve to get the attention of the female. Further on, after the female has noted the erect penis, these signals express the male’s insistence and his motivation to trigger the female into mating with him. The term "attention getter" often used for these signals therefore is misleading, which is also pointed out by Liebal and Call (2012).

^7^In language, whole chains of shifts can emerge in this way. Thus, some changes in the phonological inventories and lexicons of languages are commonly interpreted in this manner (e.g., the Great Vowel Shift for English).

^8^Here, we illustrate one of the two possible scenarios just mentioned.

^9^In the East group there is no evidence for the ground-nest gesture being used in the mating context. Given the lack of diachronic data, it is impossible to find out retroactively why this is the case. Either the ground-nest gesture never emerged in the mating context. Or it emerged and subsequently was lost, similar to the use of cushion-making by a limited number of male chimpanzees in the mating context in Mahale (Tanzania; Nishida, 1987; Boesch, 1995), which was used over several months, but never became fully established, and eventually vanished.

^10^Data collection in the Taï National Park was non-invasive and was carried out in compliance with the requirements and guidelines of the “Ministère de l’enseignement supérieure et de la recherche scientifique” and it adhered to the legal requirements of the Côte d’Ivoire. Furthermore, the regulations of the Deutsches Tierschutzgesetz (German Animal Welfare Act) and the American Society of Primatologists (ASP) principles for the ethical treatment of non-human primates were strictly adhered to.

^11^Note that also in human communication the use of additional signals and multimodality go on all the time. When agreeing, for instance, instead of simply saying “Yes.” we often add other linguistic material, for instance “yes, that would be great.” In addition, we may combine these words with affirmative gestures in different modalities like nodding (visual) or touching (tactile), or even stomping/clapping (auditory) to show extreme enthusiasm. In doing so intentionally or unintentionally, we provide the recipient(s) with information about our attitude and commitment.
